# Dental behaviour support: can we improve qualitative research on patient experience?

**DOI:** 10.1038/s41432-024-01040-4

**Published:** 2024-08-06

**Authors:** Richard D. Holmes

**Affiliations:** School of Dental Sciences, Newcastle University, Newcastle upon Tyne, NE2 4BW United Kingdom

**Keywords:** Dental psychology, Dental public health

## Abstract

**Data sources:**

Databases included Embase, Medline (via OVID) and PsycINFO (via EBSCO). Studies referenced within included review articles were additionally screened for relevance.

**Study selection:**

This review focused upon qualitative research studies and their use of dental behaviour support (DBS) tools to support dental care. Included studies were restricted to those in English and published since 1997. Screening of studies involved several authors according to pre-agreed inclusion and exclusion criteria. In the event of disagreement, a third author mediated the collaborative discussion.

**Data extraction and synthesis:**

Included studies first provided baseline study information including the type of qualitative research, the population studied and details of the type(s) of DBS under investigation. Then the qualitative data generated by each study, together with any interpretation provided by the authors, was entered onto bespoke data collection forms. A thematic synthesis approach was adopted. The authors generated new themes supported by selected quotations. The methodological quality of each included study was explored through a recognised tool and the level of confidence provided by each study was informed by the GRADE-CERQual assessment process.

**Results:**

Twenty-three studies were included. Most studies used semi-structured interviews, followed by focus groups and a small number of video diaries. For most studies, the focus was upon the dental care of children with a good proportion of these exploring dental general anaesthesia (DGA). Indeed, DGA was the most studied DBS technique. Whilst some studies explored adults’ experiences of DBS, none of the included studies centred upon medically compromised or older adults. The review authors identified five themes following data synthesis. These themes included the following areas (abridged and modified from the review paper): trust; information sharing; control and autonomy; perceived treatment success and failure of DBS techniques; and the longer-term impact of DBS techniques upon patients.

**Conclusions:**

Qualitative research has more to offer our understanding of DBS techniques and the impact they have upon patient care experience. There is a need for research to explore a wider range of DBS techniques used in isolation or in combination. Patient reported experiences of care should be considered in the development of outcome measures and any related DBS Core Outcome Set.

## A Commentary on

**Geddis-Regan A, Fisal A B A, Bird J, Fleischmann I, Mac Giolla Phadraig C.**

Experiences of dental behaviour support techniques: A qualitative systematic review. *Community Dent Oral Epidemiol* 2024; 10.1111/cdoe.12969.

**GRADE Rating:** 

## Commentary

This PROSPERO-registered, qualitative systematic review focused upon patient, carer and parental experiences of dental behaviour support (DBS)^1^. DBS may be viewed as an overarching term which includes a wide range of options from communication-based interventions and specific behaviour management techniques through to approaches including dental general anaesthesia (DGA)^2^. A wide range of DBS techniques are used regularly in the support and delivery of professional oral health care, but their evaluation has tended to focus upon trials using a wide range of quantitative outcome measures with the risk this may result in selective reporting and/or unnecessary heterogeneity^3^.

This systematic review reports a clear aim and objectives, research question, a PICOS-guided search strategy and PRISMA flow diagram. The inclusion and exclusion criteria encompass all patient groups recruited to qualitative studies since 1997. However, only studies published in English were included and there is a risk that relevant studies may not have been identified if other terminologies were used. The study population was sensibly broad, including children and adults, as well as parents and carers acting in a supportive capacity.

The 23 included studies were from high-income countries and the most studied DBS technique was general anaesthesia. Notably, sixteen of the included studies focused upon children with none centred upon medically compromised or older adults. The review authors noted that the theoretical stance, as well as cultural and contextual factors were rarely specified within the primary studies.

A strength of this qualitative systematic review was the authors’ application of the Grade-CERQual assessment tool which provides guidance for how much confidence may be placed in findings from a systematic review (or evidence synthesis) involving qualitative research^4^. Confidence in the included research studies ranged from low to high (by theme). Most themes generated by this synthesis were associated with ‘moderate’ level confidence or above, according to the authors’ application of GRADE-CerQual. The evidence synthesis ultimately led the review team to recommendations within which they ‘broadly have confidence’^1^.

Patient and stakeholder experiences of care offer unique insight and rich data to inform our knowledge about DBS techniques and how they are perceived by patients and carers. It is incumbent upon researchers to ensure that qualitative studies are designed and reported appropriately, driving up quality and rigour. There is a need for more patient-centred qualitative research and for this to expand beyond DGA in children to include experiences of a wider scope of DBS techniques across all age groups.

Practice points

- Building trust and facilitating autonomy appears to support patients’ positive experiences of dental care.
- Some dental behaviour support (DBS) techniques may exert longer-term impacts on patients extending beyond the time of their application.
